# Unconvincing evidence for peripheral biomarkers in major mental disorders

**DOI:** 10.1038/s41398-021-01355-1

**Published:** 2021-04-23

**Authors:** Klaus Munkholm

**Affiliations:** 1 Centre for Evidence-Based Medicine Odense (CEBMO) and Cochrane Denmark, Department of Clinical Research, University of Southern Denmark, Odense, Denmark; 2 Open Patient data Exploratory Network (OPEN), Odense University Hospital, Odense, Denmark

**Keywords:** Schizophrenia, Bipolar disorder, Diagnostic markers, Depression

Dear Editor,

Vast resources have been invested in research into biomarkers in mental disorders with the number of annual publications increasing ten-fold over the past two decades. Thus, I read with great interest the umbrella review by Carvalho et al. that aimed to identify peripheral biomarkers for major mental disorders supported by the most convincing evidence^1^. The authors included 110 publications with meta-analyses of a total of 162 different biomarkers across various disorders and found that only two biomarker associations met their criteria for convincing evidence: basal awakening saliva cortisol in euthymic patients with bipolar disorder compared with healthy controls and serum pyridoxal in patients with schizophrenia compared with healthy controls, respectively. However, given the authors’ criteria for grading the credibility of the evidence, even these sobering findings may be optimistic.

Carvalho et al. graded the credibility of the evidence of each association into four classes, from convincing (class I) to weak evidence (class IV), and used an additional class of “non-significant” for meta-analyses with statistically non-significant results. They classified the evidence as convincing when the meta-analyses had an estimated power >0.8 to detect an effect size (standardized mean difference) of 0.2, no large heterogeneity (i.e., *I*^2^ < 50%), a 95% prediction interval not including null, no evidence of excess significance bias, no evidence of small-study effects and significant associations at *P* < 0.005. Several of these criteria, however, rely on statistical methods that are problematic when the number of studies is small. This was the case for the two biomarkers that Carvalho et al. found to show convincing evidence—the meta-analysis for each of those biomarkers included just 5 studies each^2,3^. Specifically, statistical methods to detect small study effects, including the Egger test used by the authors^4^, have low power, which means that reporting biases cannot generally be excluded^5^. For that reason, it has been recommended that tests for funnel-plot asymmetry are not used when there are fewer than 10 studies^5,6^. Similarly, the statistical test for heterogeneity has low power when studies are small or few in number, and the uncertainty in the value of *I*^2^ is, for that and other reasons, substantial when the number of studies is small^7^. Lastly, prediction intervals, which are strongly based on the assumption of a normal distribution of effects across studies, can also be very problematic when the number of studies is small, in which case they can be spuriously wide or narrow^7^. Their use is therefore only recommended provided that the number of studies exceeds 10 and when there is no clear funnel plot asymmetry^7^.

The above methods were not only inappropriate for the two biomarkers for which Carvalho et al. found the evidence to be convincing but for most of the included meta-analyses: 225 (63%) of the 359 meta-analytic estimates included by Carvalho et al. were based on fewer than 10 studies.

In addition to these issues, the evidence criteria used by Carvalho et al. did not consider the risk of bias beyond reporting biases addressed by their tests for small-study effects and excess significance bias. As biases and confounding inherently threaten the validity of observational studies^8^, they should be of great concern when evaluating and reporting on the body of evidence for biomarkers based on observational studies; the pooling of studies, no matter how many, even when low heterogeneity is observed, does not mitigate the concerns when there is inherent bias^9^. The meta-analyses for both biomarkers considered to provide convincing evidence by Carvalho et al. were based on raw, unadjusted measurements of cortisol and pyridoxal, respectively^2,3^, and none of the studies in the pyridoxal meta-analysis and only two of five of the studies in the cortisol awakening level meta-analysis described any matching between patients and healthy controls. The studies were therefore at risk of confounding, but even if adjustment for confounding factors had been carried out, however, residual confounding would have remained a potentially serious problem^10^. Carvalho et al. assessed the methodological quality of the included meta-analyses with the AMSTAR^11^ tool, and, while not including the assessment in their evidence criteria, they described the overall methodological quality as high. However, the overall confidence in both meta-analyses found to provide convincing evidence by Carvalho et al. should likely be rated as critically low according to AMSTAR 2^12^, as they lacked a pre-registered protocol, did not provide justification of exclusion of individual studies, did not include a risk of bias assessment of individual studies and lacked consideration of the risk of bias when interpreting their results. Regardless, the quality assessment by Carvalho et al. did not have any impact on their conclusions and, importantly, Carvalho et al. did not consider the inherent limitations pertaining to confounding and other biases in their interpretation of their findings, as is often the case in reports of observational studies in psychiatry^13^.

In conclusion, the evidence presented by Carvalho et al. for any peripheral biomarker may not be all that convincing after all. Not only should evidence criteria be based on statistical tests that are appropriate for the evidence base in question, but without proper appraisal of the risk of bias, including confounding, an assessment of the certainty of the evidence for biomarkers based on observational studies conceptually lacks meaning. Given the methods and the data presented by Carvalho et al., it appears misleading to label the evidence for any peripheral biomarker in major mental disorders as convincing.
